# Supplementary material for: Genomic evidence for homoploid hybrid speciation between ancestors of two different genera
Source: Nat Commun. 2022 Apr 13;13:1987. doi: 10.1038/s41467-022-29643-4 (PMC9008057; doi:10.1038/s41467-022-29643-4)
Supplement: Supplementary file 1 — Supplementary Information [file 41467_2022_29643_MOESM1_ESM.pdf]

Genomic evidence for homoploid hybrid speciation between ancestors of two different genera

Wang *et al.*

Supplementary Note 1. Genome of *Carpinus viminea*

Sample collection, DNA extraction, and genome sequencing

Fresh leaves of a wild *C. viminea* individual were collected from Ya'an, Sichuan Province, China (102°45′E, 30°23′N). Total genomic DNA was extracted with the cetyltrimethyl ammonium bromide (CTAB) method [1]. DNA integrity was evaluated on a 0.75% agarose gel. The purity of the DNA was determined using a Nanodrop (Thermo Fisher Scientific, Waltham, MA, USA) (OD 260/280 between 1.8 and 2.0, OD 260/230 between 2.0 and 2.2) and the concentration of the DNA was determined by Qubit (Thermo Fisher Scientific, Waltham, MA, USA).

To construct the Nanopore sequencing library, the genomic DNA was used to select large fragment sizes with a BluePippin Automatic Nucleic Acid Recovery System (Sage Science, Beverly, MS, USA). The DNA fragments were end-repaired and the adapters were ligated using a Ligation Sequencing Kit (LSK) 109. The library was sequenced on a PromethION DNA sequencer (Oxford Nanopore, Oxford, UK). Then we performed Guppy for basecalling (mean_qscore_template ≥ 7) [2] and obtained a total of 40.55 Gb clean reads (~ 113.24×) with an N50 read length of 31.73 Kb (Supplementary Table 1). The clean reads were used to assemble the genome.

Paired-end Illumina libraries with insert size of ~ 350 bp were constructed using standard manufacture's protocols and then sequenced on an MGISEQ-2000 platform (Illumina, San Diego, CA, USA). Reads with adapter, PCR duplicates, low quality (quality value ≤ 5, low-quality base > 50%) and/or high N content (> 10%) were removed. A total of 55.51 Gb (~ 155.00×) 150-bp paired clean reads were generated to estimate genome size and correct the genome (Supplementary Table 1).

In addition, a Hi-C library was constructed using young leaf tissue from the same *C. viminea* individual following Louwers et al. [3] for chromatin extraction and digestion, DNA ligation, purification, and fragmentation. Then, it was sequenced with an Illumina HiSeq 4000 platform (Illumina, San Diego, CA, USA) and a total of 62.15 Gb (~ 170.75×) 150-bp paired reads were generated after adapter trimming and quality filtration (Supplementary Tables 1 and 2). These reads

were later applied to extend the contiguity of the genome assembly to the chromosomal level.

We also collected four fresh tissue samples (leaf, flower, bud, and twig) from the same *C. viminea* individual for total RNA sequencing. For each tissue, high-quality total RNA was extracted following a modified CTAB method [4], and was then used to construct a cDNA library with an NEBNext Ultra RNA Library Prep Kit for Illumina (NEB). The raw reads were generated by an Illumina HiSeq 4000 platform (Illumina, San Diego, CA, USA), and were then filtered by Trimmomatic [5] with the options: ILLUMINACLIP:TruSeq3-PE-2.fa:2:30:10 LEADING:3 TRAILING:3 SLIDINGWINDO W:4:15 MINLEN:50 TOPHRED33. Finally, a total of 30.54 Gb RNA-Seq clean reads were obtained for subsequent analyses (Supplementary Table 1).

Genome size and heterozygosity estimation

A 17-mer frequency distribution analysis was performed using the clean data from Illumina short reads, via Jellyfish [6], with the highest peak occurring at a depth of 64 (Supplementary Fig. 2). The estimated genome size was 358.11 Mb, and the heterozygosity rate for the genome was 1.25% (Supplementary Table 3).

Genome assembly

The Nanopore long reads were corrected using NextDenovo (https://github.com/Nextomics/NextDenovo) (read_cuoff = 3k, seed_cutoff = 25k, blocksize = 2g) and *de novo* assembled using SmartDenovo (https://github.com/ruanjue/smartdenovo) (wtpre -J 3000, wtzmo -k 21 -z 10 -Z 16 -U -1 -m 0.1 -A 1000, wtclp -d 3 -k 300 -m 0.1 -FT, wtlay -w 300 -s 200 -m 0.1 -r 0.95 -c 1). The contigs were corrected and polished with the Illumina reads using Pilon [7] for three rounds. We obtained a 375.17 Mb genome sequence with a contig N50 of 4.67 Mb. Pseudo-chromosomes of *C. viminea* were then constructed using the Hi-C library. The Hi-C clean reads were mapped to the assembled genome using bowtie2-2.3.2 [8]. Valid reads were selected after removal of duplicates, and quality was assessed using HiC-Pro [9] (Supplementary Tables 4 and 5). Then we used LACHESIS [10] with the parameters CLUSTER MIN RE SITES = 100, CLUSTER MAX LINK DENSITY = 2.5, CLUSTER NONINFORMATIVE RATIO = 1.4, ORDER MIN N RES IN TRUN = 60, and ORDER MIN N RES

IN SHREDS = 60 to cluster, reorder, and orientate the corrected contigs into pseudochromosomes by examining their interactions in the Hi-C heatmap.

Finally, a total of 241 contigs (constituting 99.34% of the genome assembly) were successfully anchored onto eight chromosome groups. The final chromosome-scale genome was 372.72 Mb in length with a contig N50 of 4.31 Mb, a scaffold N50 of 42.12 Mb, and a maximum pseudochromosome length of 68.30 Mb (Supplementary Fig. 3 and Supplementary Tables 6 and 7).

Repeat annotation

Homolog-based and *de novo* approach pipelines were used to annotate the repeat sequences in the *C. viminea* genome. For the homology-based method, RepeatMasker v.4.0.7 [11] was applied to search for transposable elements based on the Repbase database [12]. For the *de novo* method, RepeatModeler v.1.0.10 (http://www.repeatmasker.org) was used to construct a *de novo* repeat sequence library, and RepeatMasker was used to identify repeat sequences against this library. Finally, a total of 121.88 Mb repetitive elements (32.43% of the assembly) were identified in the *C. viminea* genome, including retroelements (13.78%), DNA transposons (2.25%) and unclassified elements (12.97%) (Supplementary Table 8).

Gene prediction

We assembled transcriptomes with Trinity v.2.4.0 [13] based on *de novo* and genome-guided strategies. PASA v.2.1.0 (Program to Assemble Spliced Alignments) [14] was run to align the transcripts to the assembled genome to carry out ORF prediction and gene prediction. To train the HMM model for Augustus [15], we then extracted complete, multiexon genes, removed redundant high-identity genes (cut-off all-to-all identity of 70%), and thus generated the best candidate and low-identity gene models for training. We aligned the RNA-seq data to the hard-masked genome assembly with HISAT2 [16] and used bam2hints packaged in Augustus to generate an intron hint file. This hint file was used to carry out *ab initio* gene prediction by Augustus v.3.2.3.

For homology prediction, the reference protein sequences of *Arabidopsis thaliana* [17], *Betula pendula* [18], *Ostrya rehderiana* [19], and *Carpinus fangiana* [20] were downloaded and aligned against the genome

using TBLASTN v.2.2.31 [21] and searched with an E value threshold of 1E-05. After filtering low-quality results, gene structure was predicted using GeneWise v.2.4.1 [22]. We combined the results from PASA, Augustus, and GeneWise to generate the final protein-coding gene set using EVidenceModeler (EVM) v.1.1.1 [23] with the following weights: Augustus 1, GeneWise 5, and PASA 10. To obtain the untranslated regions (UTRs) and alternatively spliced isoforms, we used PASA to update the GFF3 file for two rounds and so obtained the final gene models. Finally, a total of 26,621 protein-coding genes were predicted in the genome of *C. viminea* (Supplementary Table 9).

Functional annotation

We annotated the functions of the predicted genes by BLAST+ v.2.2.31 [21] with a cut-off E value of 1E-05 and a maximum target sequences number of 20, against public databases including Swiss-Prot [24], TrEMBL [24] and NCBI non-redundant protein (NR) [25] databases. Best-hit BLAST results were then used to define gene functions. We used InterProScan v.5.25-64.0 [26] to identify motifs and domains by matching against public databases. We added Gene Ontology (GO) [27] annotations by using the Blast2GO v.4.1 pipeline [28] based on the blast results and combined them with InterPro GO entries. We submitted the predicted proteins to the KEGG (Kyoto Encyclopedia of Genes and Genomes) Automatic Annotation Server (KAAS) [29] to obtain KO numbers for KEGG pathway annotation. A total of 25,929 genes (97.40% of all predicted protein-coding genes) were successfully annotated against at least one database (Supplementary Table 10).

Technical validation

Assessment of the genome assembly

The completeness of the genome assembly was evaluated using Benchmarking Universal Single-Copy Orthologs (BUSCO) [30] (v3, embryophyta_odb10 [31]). Among 1,375 plant-specific orthologs, 1,332 (96.87%) were identified as “Complete BUSCOs” in the assembly, and only 24 (1.75%) of them were missing (Supplementary Table 11). The evaluation was also performed based on the RNA-Seq reads from four different tissues. The reads were mapped to the assembled genome using HISAT2. The mapping ratios were 97.68% (flower), 93.33% (leaf), 97.81% (bud) and 96.58% (twig)

(Supplementary Table 12). These results demonstrated high completeness for the genome assembly.

We then assessed the accuracy of clustering for eight chromosomes based on the Hi-C data. We split the anchored genome into “bins”, each with a size of 100 Kb, and detected their interaction signals by counting the number of Hi-C read pairs covered between any two of the “bins”. In a heatmap of divided “bins”, we found eight clearly distinct groups, indicating a high accuracy for the chromosome assembly (Supplementary Fig. 3).

Improvement of gene prediction

To validate the results, we applied a robust pipeline for gene prediction. Before annotation, repeats in the genome were identified and masked. Protein-coding genes were predicted using different approaches, including *ab initio*, homology-based, and transcriptome-based strategies, and merged with EVM. Candidate genes were then removed if: (1) internal stop codons or partial codons were present; (2) the initiation codon or stop codon were missing; (3) their CDS lengths were less than 150 bp; (4) gene regions overlapped with one another. Finally, untranslated regions (UTRs) were identified by PASA for two rounds.

Assessment of the gene prediction

The completeness and accuracy of the protein-coding genes were also evaluated based on BUSCO and RNA-Seq reads respectively. For BUSCO analysis, among 1,375 plant-specific orthologs, 1,258 (91.49%) were identified as “Complete BUSCOs” in the proteins, and only 48 (3.49%) were missing (Supplementary Table 11). Then we mapped the reads to the coding sequences (CDS) of genes by HISAT2. A total of 24,711 genes (92.83% of all genes) were covered at least once (Supplementary Table 12). The high percentage of “Complete BUSCOs” and the high mapping ratio for RNA-Seq reads indicated high-quality gene prediction.

Supplementary Note 2. Hi-C sequencing and chromosome anchoring for the *Ostrya rehderiana* genome

To further extend the contiguity of the genome assembly, we performed Hi-C sequencing and chromosome anchoring for *O. rehderiana* following the pipeline described in Supplementary Note 1.

Sample collection and Hi-C sequencing

A fresh sample of *O. rehderiana* was collected from Tianmu Mountain, Zhejiang Province, China (119°27′E, 30°20′N) for Hi-C sequencing. A total of 51.51 Gb (~ 140.65×) 150-bp clean paired reads were generated for improvement of the *O. rehderiana* genome (Supplementary Table 13).

Chromosome anchoring

Our previously published *O. rehderiana* genome was 366.20 Mb in length, with 1,534 scaffolds (≥2000 bp), a scaffold N50 of 2.31 Mb, and a contig N50 of 21.96 Kb [19]. We mapped the Hi-C clean reads to the raw assembly using Bowtie2-2.3.2 [8]. The mapped reads were analyzed and assessed using HiC-Pro [9] (Supplementary Tables 14 and 15). LACHESIS [10] was used to perform chromosome anchoring described in Supplementary Note 1. Finally, a total of 337 scaffolds were anchored onto eight chromosome groups. The final chromosome-scale genome was 344.69 Mb in length, occupying 93.22% of the raw genome assembly (Supplementary Fig. 4 and Supplementary Table 16).

Supplementary Note 3. Genome features and comparative genomic analyses

Genome features

For *C. viminea* assembly, GC (guanine-cytosine) content, repeat density, and gene density were summarized by a 500 Kb non-overlapping sliding-window respectively. The synteny blocks (involving ≥ 5 collinear genes) within *C. viminea* genome were identified by MCScanX [32]. Genome features were visualized by Circos [33].

Phylogenomic species tree

The comparative genomic analyses were performed with the representative species of different Betulaceae genera, including *C. viminea*, *C. fangiana* [20], *O. rehderiana*, *Ostryopsis davidiana* [34], *Corylus mandshurica* [35], and *Betula pendula* [18]. *Casuarina equisetifolia* [36] and *Juglans regia* [37] were used as the outgroups. A total of 2,562 strictly orthologous gene groups (1:1:1:1:1:1:1:1) were identified between these eight species by OrthoFinder [38] with the default parameters. For each pair of orthologs, their coding sequences (CDS) were aligned by PRANK [39] with the parameter: -codon. After the removal of orthologs with short aligned regions (< 300 bp) or low alignment ratio (< 50%), 1,970 strictly orthologous gene groups were used to reconstruct the phylogenomic tree. We extracted their 4DTv sites and generated a concatenated matrix. RAxML [40] was used to construct a maximum likelihood (ML) tree under the GTRGAMMA model with 100 bootstraps. *J. regia* served as the outgroup.

Collinearity analysis

To explore the evolution of chromosome ploidy and detect chromosome-level recombination events, we performed collinearity analysis using MCScanX [32] between the six Betulaceae species (involving ≥ 20 collinear genes) and within each of them (involving ≥ 5 collinear genes). Within each species, the *K*s (synonymous substitutions per synonymous site) values of each paralogous gene pair were calculated by the script "add_ka_and_ks_to_collinearity.pl" in the MCScanX package. The LAST [41] pipeline was also performed between *C. viminea*, *C. fangiana*, and *O. rehderiana* based on their whole

genome sequences with the recommended parameters. The results were visualized with the script "last-dotplot" in the LAST package (Supplementary Fig. 5).

Supplementary Note 4. Hybridization test based on *de novo* genome sequences

We performed hybridization tests among the three species, *C. viminea*, *C. fangiana* [20], and *O. rehderiana* [19], based on their *de novo* genome sequences.

Gene family identification

We used the protein sequences of *C. viminea*, *C. fangiana*, *O. rehderiana*, and *Ostryopsis davidiana* (as outgroup, since it has the best quality among the published genomes of three *Ostryopsis* species) [34] for gene family identification. The longest transcript of each gene was extracted. OrthoFinder [38] was employed with the default parameters. Finally, a total of 106,376 genes were classified into 28,122 gene families for the four species. Among them, we identified 7,468 strictly orthologous gene groups (1:1:1:1).

Phylogenetic analysis

We performed phylogenetic analyses based on the 7,468 strict ortholog groups (1:1:1:1). For each pair of orthologs, PRANK [39] was used to align their protein-coding sequences (CDS) with the parameter: -codon. We then removed orthologs with short aligned regions (< 300 bp) or low alignment ratio (< 50%). A total of 6,321 pairs of orthologs were retained for subsequent analysis. We extracted the 4DTv sites to construct phylogenetic trees for each pair of orthologs. A maximum likelihood (ML) tree was constructed by RAxML [40] under the GTRGAMMA model with 100 bootstraps. *Ostryopsis davidiana* was set as the outgroup. Using the 6,321 produced gene trees, ASTRAL [42] was used to estimate the species tree under a multi-species coalescent model. The branch lengths of the species tree so generated were in coalescent units. Then phylogenetic trees with low bootstrap values (< 50) were further removed. Finally, we obtained a total of 4,769 phylogenetic trees with high-confidence support values, comprising three different topologies as shown in Fig. 3a,b.

The exclusion of incomplete lineage sorting (ILS) interference by simulation

We simulated the effects of ILS on the composition of different topologies for gene trees. We used DendroPy [43] to simulate the gene trees under the ILS scenario using the species tree previously

obtained as the input data. For each time, we simulated 4,769 gene trees (equal to the number of previously obtained phylogenetic trees with high-confidence support values) and counted the number of different phylogenetic topologies. We calculated the ratio of the two lower-number phylogenetic topologies (Topology III and Topology II in Fig. 3b). In theory, if there is only the effect of ILS with no hybridization event, the expected value of this ratio would be 1.

The simulation was performed for a total of 10,000 replicates. We summarized the ratios of Topology III and Topology II from each time of simulation as the null distribution. Based on the 4,769 high-confidence gene trees previously obtained, we also calculated the actual ratio of Topology III and Topology II as the observed value. A two-tailed one-sample Student's *t*-test was employed to examine the significance of the difference between the observed value and the null distribution.

Divergence time estimation based on *K*s values

To infer the time scale of the hybridization event, we estimated the times of divergence between the three species based on the distributions of *K*s (synonymous substitutions per synonymous site) values and a secondary calibration. First, we identified single-copy (1:1) orthologs between each pair of the four species: *C. viminea*, *C. fangiana*, *O. rehderiana*, and *Betula pendula* [18]. We calculated the *K*s values for each pair of single-copy orthologs and obtained the *K*s distributions and the corresponding *K*s peak values for each pair of the species (Fig. 2d). We found that the *K*s peak values for *Betula pendula* and each of the other three species were all in the range 0.11-0.12. The *K*s peak values for *O. rehderiana* and each of the two *Carpinus* species were in the range 0.04-0.05. The *K*s peak value for *C. viminea* and *C. fangiana* was between 0.03 and 0.04.

Based on the formula: $T=Ks/2\mu$ (where T indicates the time of divergence between two species, *K*s indicates the *K*s peak value, and μ indicates the mutation rate), it follows: $T_1/T_2=(Ks_1/2\mu_1)/(Ks_2/2\mu_2)$. Supposing that different lineages have similar mutation rates, we have $T_1/T_2=Ks_1/Ks_2$. Based on previous studies [44,45], the divergence time between *Betula pendula* and the other three species was set as ~71 million years ago (mya). From this, the genera *Ostrya* and *Carpinus* were estimated to diverge at 23-33 mya and the divergence time between sects. *Carpinus* and *Distegocarpus* was dated to 17-26 mya (Fig. 2c,d).

Supplementary Note 5. Population re-sequencing, read mapping, and variant calling

To explore more genetic detail, we sampled population materials, re-sequenced their genomes, and performed read mapping and variant calling.

Population materials

We collected 47 individuals (including a total of 21 species) from three different lineages for population genomic re-sequencing: 10 individuals (from 10 species) of sect. *Carpinus*, 27 individuals (from 3 species) of sect. *Distegocarpus*, 7 individuals (from 7 species) of *Ostrya*, and 3 individuals (from 1 species) of *Ostryopsis* as outgroup. Except for the outgroup, all the sampled individuals were selected from different populations (one individual per population). The samples of sect. *Carpinus* and *Ostrya* covered all the major species of these two lineages. The samples of sect. *Distegocarpus* covered all 3 species in this lineage and almost the whole of their distributions in the wild (Fig. 2a and Supplementary Data 2).

For each sample, we extracted total genomic DNA by the CTAB method [1] and assessed DNA quality by gel electrophoresis, Nanodrop, and Qubit. Paired-end Illumina libraries were then constructed following the standard pipeline. An Illumina HiSeq platform (Illumina, San Diego, CA, USA) was used to generate the raw reads for each sample. Reads with adapter and PCR duplicates were removed. Low-quality reads (> 65% low-quality bases with a PHRED-like score < 8 or > 5% 'N' content) were also removed. Finally we obtained an average of ~ 10.94 Gb (> 25×) clean bases for each sequenced sample (Supplementary Data 4).

Read mapping

Because the quality was higher (see in Supplementary Notes 1 and 2) and more sequenced individuals of the genus *Carpinus* would produce a better performance (Supplementary Data 3), *C. viminea* genome was selected as the reference genome. We mapped the filtered reads for each sample to the reference genome, by BWA-MEM [46] with recommended parameters. Only the primary alignments

were retained for subsequent analyses. Next, using SAMtools [46], we sorted the mapped reads and removed duplicated reads. Finally, we obtained BAM files for each sample, with an average mapping ratio of 88.93% and a more than 20× mapping depth, covering 75.57% of the reference genome (Supplementary Data 4). The BAM files thus generated were used for variant calling.

SNP calling

We applied the pipeline corresponding to GATK best practice to identify SNP variants at the population level. GATK HaplotypeCaller [47] was first used to detect the variants for each sample. Then the variants identified for each sample were merged by GATK GenotypeGVCFs [47]. A set of robust criteria was then applied to filter out raw datasets: (1) indels and their surrounding regions (5 bp regions around them) were removed; (2) only biallelic SNPs were retained; (3) hard filtering by GATK VariantFiltration was applied (--filterExpression "QUAL<30 || MQ<40.0 || QD<2.0 || FS>60.0 || ReadPosRankSum <-8.0 || MQRankSum < -12.5"); (4) only SNPs supported by 0.33-3 times the corresponding individual's average depth were retained; the others were treated as missing data; (5) if the missing data from each site were more than 20% in any lineage of sect. *Carpinus*, sect. *Distegocarpus*, or *Ostrya*, this SNP variant would be removed. Finally, we obtained a high-quality SNP set, containing 6,244,030 biallelic SNPs with the outgroup and 6,302,136 biallelic SNPs without the outgroup.

Indel detection

We also detected indels for each sample. The indels were extracted from the raw merged file previously generated by GATK GenotypeGVCFs. Then, we applied a set of robust criteria to filter the dataset: (1) if different indels shared overlapping regions, they were removed; (2) indels shorter than 5 bp were removed; (3) hard filtering by GATK VariantFiltration was applied (--filterExpression "QUAL<40 || QD<2.0 || FS>200.0 || ReadPosRankSum <-20.0"); (4) indels supported by fewer than 3 reads or more than 300 reads were treated as missing data; (5) only indels with less than 20% missing data in each lineage of sect. *Carpinus*, sect. *Distegocarpus*, and *Ostrya* were retained. Finally, a total of 443,792 indels (with outgroup) were identified.

Supplementary Note 6. Hybridization test based on population genomic data

To explore the hybridization event in greater depth, we employed hybridization tests using the population genomic data.

Phylogenetic analysis

To determine the population-level phylogenetic relationships and examine whether they are identical with those produced by single genomes, we performed population-level phylogenetic analysis using the previously generated 6,244,030 biallelic SNPs. RAxML [40] was employed with 100 bootstraps under the GTRGAMMA model. *Ostryopsis* individuals were set as the outgroup.

HyDe analyses based on indels

The status of SNP sites from different lineages may conflict with the real evolutionary relationships due to the effects of homoplasy (parallel or convergent evolution), especially for ancient diverged lineages [48–50]. Long indels (≥ 5 bp) are a rare type of genomic event, with much lower frequency of homoplasy than shorter indels and SNPs [48–50]. Long indels are therefore a valid tool with which to evaluate ancient evolutionary relationships.

We used HyDe [51] to test hybridization scenarios based on the 443,792 long indels (≥ 5 bp) previously generated. To reduce the bias produced by detecting indels based on Illumina data and because recombination of each indel could be treated as an independent event, we discarded length information about the indels and set each of them to have the same weight. Thus, for the input file, the indels were transformed into A/T 'pseudo-alleles', indicating their status of present/absent. The individuals were classified into four groups: sect. *Carpinus*, sect. *Distegocarpus*, *Ostrya*, and *Ostryopsis*. *Ostryopsis* individuals were set as the outgroup. The analysis was performed at the population level following the HyDe user guide [51]. The program "run_hyde_mp.py" was applied with the parameters "-i input_file -m map_file -o outgroup -n 47 -t 4 -s 443792 -q -tr triples_file".

ABBA-BABA test (*D*-statistic)

Using the previously transformed data for the 443,792 indels, we also performed an ABBA-BABA test (*D*-statistic) [52–55] using the software Dsuit [56]. The "Dtrios" program packaged in Dsuit was run with default parameters. The analysis was performed at the population level, with sect. *Carpinus*, sect. *Distegocarpus*, *Ostrya*, and *Ostryopsis* as P_1, P_2, P_3, and O, respectively.

Hybridization test based on inter-group fixed indels

Jiang et al. [50] have developed an approach with which to examine the ancient homoploid hybrid speciation (HHS) hypothesis. Based on the single genomes of three species, long indels (≥ 5 bp) can be detected in the homologous regions and are further classified into two classes: ancestral variations (AVs) and phylogenetically informative variations (PIVs). AVs and PIVs are classified based on their times of occurrence. AVs occurred before the differentiation of all species. PIVs occurred after the first species differentiated and before the last one. If significant PIV signals can be detected between the assumed hybrid species and each parental species, the HHS assumption would be validated with elimination of introgression and ILS.

However, using a single genome of the studied species might yield misleading results caused by introgression in a few individuals rather than all offspring. To avoid such errors, we modified the method and applied it to the population-level data to include all hybrid offspring.

To do so, we performed the analysis using the 443,792 long indels (≥ 5 bp) previously identified. We divided the individuals into four groups: sect. *Carpinus* (denoted "C"), sect. *Distegocarpus* (denoted "D"), *Ostrya* (denoted "O"), and *Ostryopsis* (as outgroup) (denoted "A"). To exclude interference from introgression, a total of 60,487 indels fixed in each of the lineages were retained and recorded for subsequent analysis. For each indel, the status (presence or absence) in *Ostryopsis* (outgroup) was recorded as "0", while the opposite status as "1". AVs and PIVs were identified based on the patterns of sharing of each indel. For AVs, in the order "A|C|D|O", "0|0|0|1" were identified as "CDA" shared indels, "0|0|1|0" as "COA" shared indels, and "0|1|0|0" as "ODA" shared indels. For PIVs, "0|1|1|0" were identified as "CD" shared indels, "0|1|0|1" as "CO" shared indels, and "0|0|1|1" as "OD" shared indels.

Then we detected PIV signals using the AVs and PIVs identified. According to the hypothesis in Jiang et al. [50], if $P_{CDA}/P_{(CD+CDA)}$ is less likely than $P_{COA}/P_{(CO+COA)}$, there is a significant number of PIV signals in "CD"; if $P_{ODA}/P_{(OD+ODA)}$ is less likely than $P_{COA}/P_{(CO+COA)}$, there is a significant number of PIV signals in "OD"; and if significant PIV signals are detected in both "CD" and "OD", the HHS model of C(D)O should be accepted, in other words "D" is a homoploid hybrid originating from "C" and "O".

Supplementary Note 7. Positively selected genes and hybrid signal detection on genes

We identified positively selected genes (PSGs) and genes harboring the hybrid signals because of hybrid recombination based on the re-sequenced individuals (using 6,302,136 biallelic SNPs without an outgroup).

For the PSGs, all individuals were divided into two sets in two different ways: (1) firstly, we treated sects. *Distegocarpus* (hybrid lineage) and *Carpinus* as one group (Group 1) and compared it with *Ostrya* (as Group 2); (2) secondly, we treated sect. *Distegocarpus* and *Ostrya* as one group (Group 1) and compared it with sect. *Carpinus* (Group 2). For each grouping approach, PSGs were identified following the method in Wang et al. [34]. First, the Hudson-Kreitman-Aguadé (HKA) test [57] was performed. For each gene, the number of polymorphic sites (SNPs) in Group 1 (termed "A") and the number of fixed differences (the SNPs with $F_{ST} > 0.95$) between Group 1 and Group 2 (termed "B") were recorded. Genome-wide A and B values were calculated as the sum of A and B values across all genes analyzed. The null hypothesis A(gene)/B(gene)=A(genome-wide)/B(genome-wide) was tested by a Pearson's chi-square test on the 2 × 2 contingency table. Moreover, we counted the fixed mutation sites that were non-synonymous between Group 1 and Group 2. The final set of PSGs was identified based on three criteria: (1) significant *P*-values (≤ 0.01, after Yates' correction for continuity) in HKA tests; (2) the number of fixed non-synonymous mutation sites ranked in the top 2.5% of all genes tested; (3) phylogenies with sect. *Distegocarpus* individuals deriving mainly from one parental lineage. Finally, we identified a total of 218 PSGs in sect. *Distegocarpus* derived from sect. *Carpinus* (Supplementary Data 5), and 73 PSGs in sect. *Distegocarpus* derived from *Ostrya* (Supplementary Data 6).

Then hybrid signals were detected according to whether the genes were positively selected with both grouping methods: sects. *Distegocarpus* (hybrid lineage) and *Carpinus* as one group compared with *Ostrya*, and sect. *Distegocarpus* and *Ostrya* as one group compared with sect. *Carpinus*. When detecting hybrid signals, we considered only two criteria (a significant *P*-value in an HKA test and the number of fixed non-synonymous mutation sites ranking in the top 2.5%), taking no account of

phylogenies. We also aligned the gene sequences to check whether sect. *Distegocarpus* contains fixed mutations from the other two parental lineages because of hybrid recombination. Finally, a total of 19 genes with significant hybrid signals were identified (Supplementary Data 7).

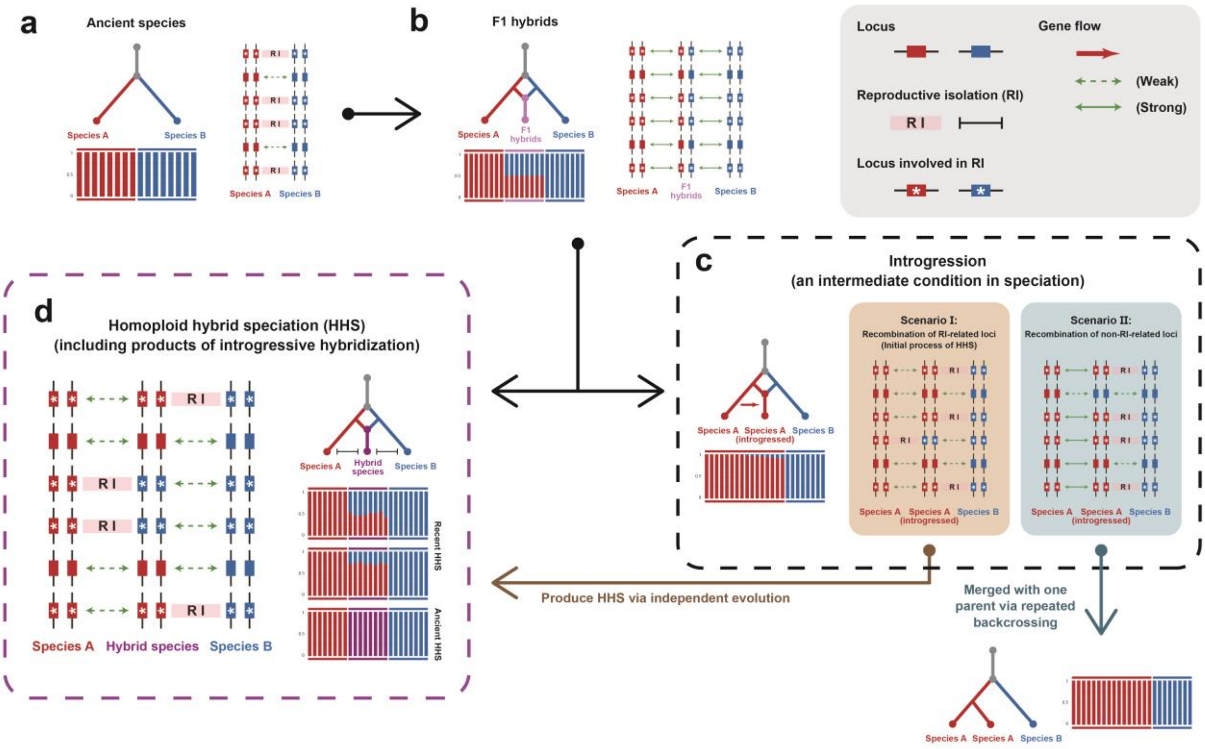

Supplementary Figure 1. Homoploid hybrid speciation (HHS) and its distinction from other hybridization outcomes. **a** Divergence of ancient species. **b** F1 hybrids. **c** Introgression. **d** HHS (arising from both F1 or backcrossing hybrids with equal or unequal genomic contributions from two parents). In (c), introgression comprises two scenarios: recombination of RI-related loci (Scenario I, also called introgressive hybridization) and recombination of non-RI-related loci (Scenario II). Scenario I can be also defined the initial HHS process.

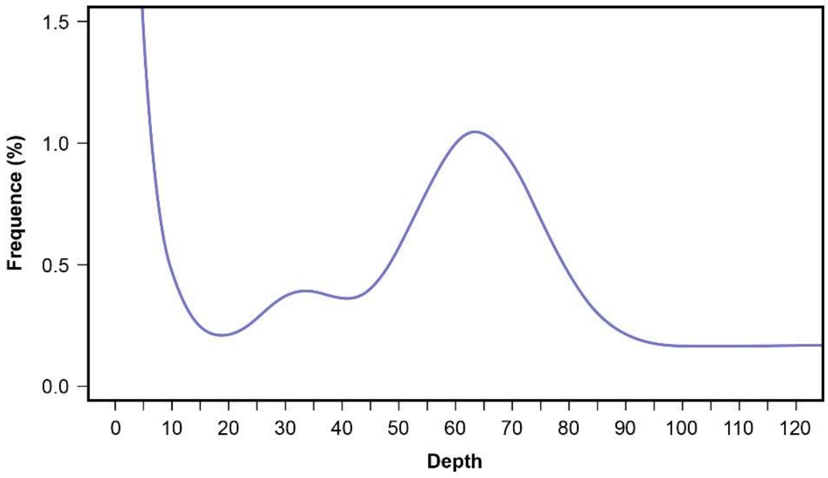

Supplementary Figure 2. 17-mer frequency analysis for genome of *Carpinus viminea*.

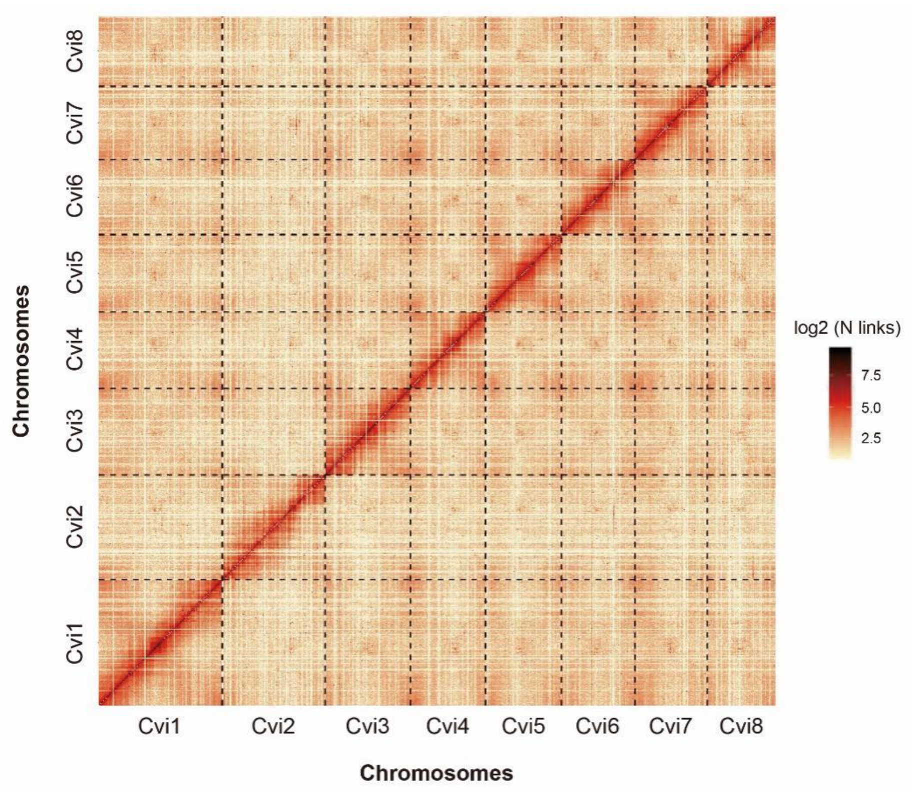

Supplementary Figure 3. The chromatin interactions of *Carpinus viminea* at 100 Kb resolution.

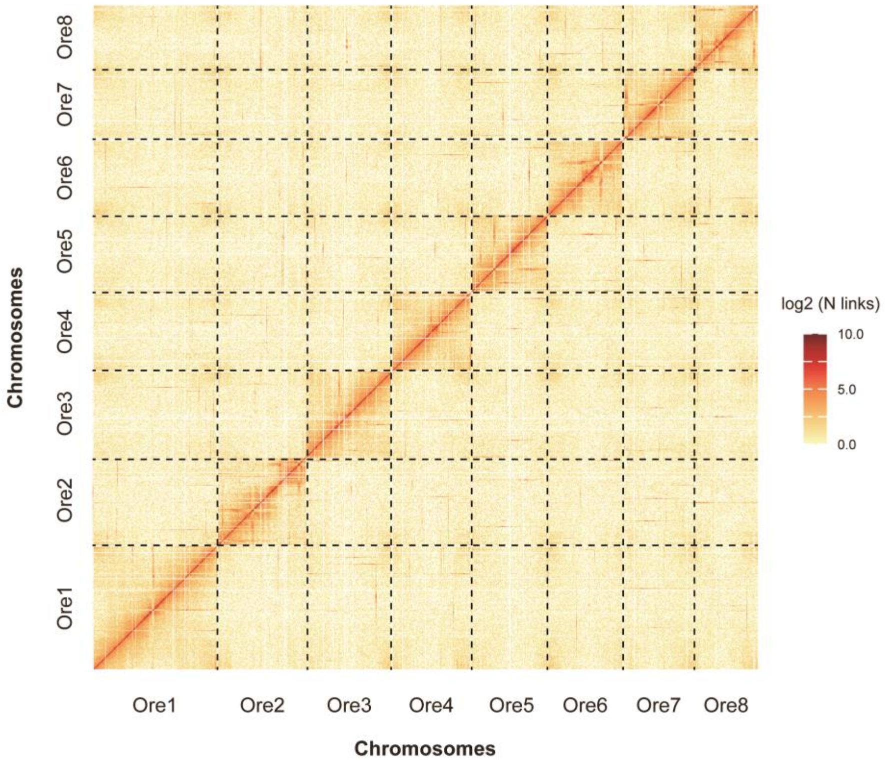

Supplementary Figure 4. The chromatin interactions of *Ostrya rehderiana* at 100 Kb resolution.

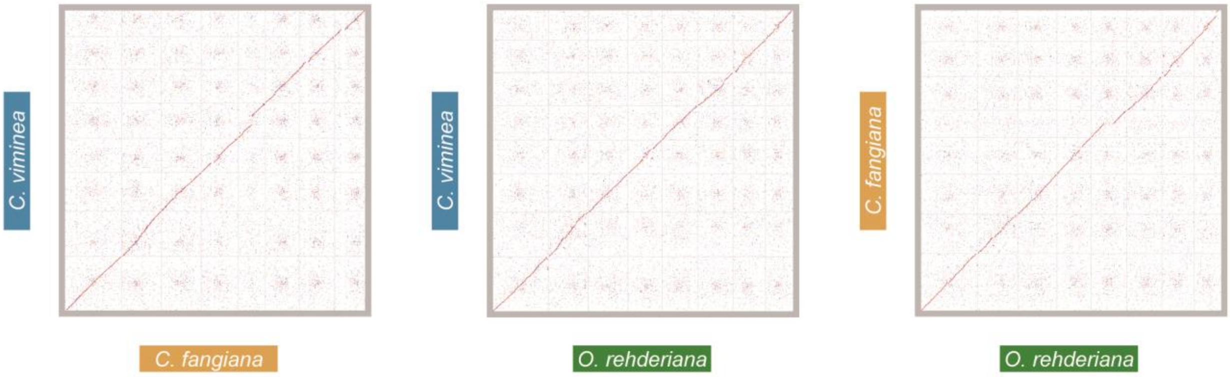

Supplementary Figure 5. Collinearity analysis at the chromosome level by LAST.

Supplementary Table 1. Summary of sequencing data generated in this study.

Library type	Platform	Read length	Clean reads number	Clean base	Coverage[a]	Application
Nanopore	PromethION	31,726 bp (N50)	1,615,333	40.55 Gb	113.24×	Genome assembly
Short reads	MGISEQ-2000	2 × 150 bp	2 × 185,027,829	55.51 Gb	155.00×	Genome survey and base-level correction
Hi-C	HiSeq 4000	2 × 150 bp	2 ×203,822,340	62.15 Gb	170.75×	Chromosome construction
RNA-Seq	HiSeq 4000	2 × 150 bp	2 × 101,807,714	30.54 Gb	—	Genome annotation

[a] Depth was calculated under the estimation of a genome size of 358.11 Mb.

Supplementary Table 2. Summary of the Hi-C reads from the genome of *C. viminea*.

**Sample**	***C. viminea***
Raw Paired-end Reads	413,987,030
Clean Paired-end Reads	407,644,680
Clean Bases (bp)	59,999,478,174
Clean Paired-end Reads Rate	96.6%
Clean Q30 Bases Rate	92.8%

Supplementary Table 3. Estimation of *C. viminea* genome size based on the 17-mer method.

K-mer	K-mer number	K-mer depth	Genome size	Heterozygosity rate
17	22,919,123,180	64	358.11 Mb	1.25%

Supplementary Table 4. Mapping of Hi-C reads in the genome of *C. viminea*.

**Sample**	***C. viminea***
Clean Paired-end Reads	203,822,340
Unmapped Paired-end Reads	10,719,076
Unmapped Paired-end Reads Rate	5.3%
Paired-end Reads with Singleton	36,326,992
Paired-end Reads with Singleton Rate	17.8%
Multi Mapped Paired-end Reads	36,071,290
Multi Mapped Ratio	17.7%
Unique Mapped Paired-end Reads	120,704,982
Unique Mapped Ratio	59.2%

Supplementary Table 5. Mapping features of Hi-C reads in the genome of *C. viminea*.

**Sample**	***C. viminea***
Unique Mapped Paired-end Reads	120,704,982
Dangling End Paired-end Reads	4,516,001
Self Circle Paired-end Reads	3,057,201
Dumped Paired-end Reads	19,368,210
Valid Paired-end Reads	92,799,018
Valid Rate	76.88%

Supplementary Table 6. Summary of *C. viminea* genome assembly.

**Type**	***C. viminea* assembly**
Assembly size (bp)	372,715,291
Gap length (bp)	23,300
Number of scaffolds	8
Max. scaffold length (bp)	68,300,551
Scaffold N50 size (bp)	42,119,730
Scaffold N90 size (bp)	38,314,214
Number of contigs	241
Max. contig length (bp)	11,177,027
Contig N50 size (bp)	4,306,997
Contig N90 size (bp)	923,875
GC content	35.74%

Supplementary Table 7. Summary of chromosome groups in the genome of *C. viminea* inferred using Hi-C data.

Chromosome groups	No. of contigs	Size of contigs (bp)
Cvi1	30	68,300,551
Cvi2	72	54,934,320
Cvi3	28	46,964,676
Cvi4	20	41,534,218
Cvi5	22	42,119,730
Cvi6	23	40,594,379
Cvi7	25	39,953,203
Cvi8	21	38,314,214
Total	241	372,715,291

Supplementary Table 8. Classification of repetitive elements annotated in the genome of *C. viminea*.

Element type	No. of elements	Length occupied (bp)	Percentage of genome
DNA	16,376	8,376,972	2.25%
LTR	37,840	40,674,664	10.91%
LINE	9,562	10,456,561	2.81%
SINE	1,382	231,608	0.06%
Low complexity	61,629	3,071,437	0.82%
Satellite	222	70,239	0.01%
Simple repeats	254,338	9,852,701	2.64%
Unclassified	164,750	48,361,713	12.97%
Total	546,099	120,881,910	32.43%

Supplementary Table 9. Summary of protein-coding genes predicted in the genome of *C. viminea*.

**Type**	***C. viminea***
No. of protein-coding genes	26,621
No. of transcripts	28,515
Average exon size per transcript (bp)	1,642.06
Average coding sequence (CDS) size per transcript (bp)	1,248.23
Average intron size per transcript (bp)	3,231.51
Average exon number per transcript	5.83
Average exon size (bp)	281.77

Supplementary Table 10. Summary of functional annotation of protein-coding genes in the *C. viminea* genome.

**Database**	**No. of annotated genes**	**Percentage**
GO	23,501	88.28%
KEGG	11,256	42.28%
InterPro	25,633	96.29%
Swiss-Prot	20,459	76.85%
TrEMBL	24,378	91.57%
NR	24,271	91.17%
Annotated[a]	25,929	97.40%

[a] At least one match in either of database above.

Supplementary Table 11. BUSCO analysis of *C. viminea* genome assembly and protein-coding genes.

Type	Genome		Proteins	
	Number	Percent	Number	Percent
Complete BUSCOs	1,332	96.87%	1,258	91.49%
Complete single-copy BUSCOs	1,306	94.98%	1,145	83.27%
Complete duplicated BUSCOs	26	1.89%	113	8.22%
Fragmented BUSCOs	19	1.38%	69	5.02%
Missing BUSCOs	24	1.75%	48	3.49%
Total BUSCO groups searched	1,375		1,375	

Supplementary Table 12. Assessment of *C. viminea* genome assembly and gene prediction based on RNA-Seq reads.

Tissue	Genome[a]	Proteins	
		Number[b]	Percentage
Flower	97.68%	22,593	84.87%
Leaf	93.33%	20,592	77.35%
Bud	97.81%	22,450	84.33%
Twig	96.58%	22,519	84.59%
Average/All	96.35%	24,711	92.83%

[a] The mapping ratio for the RNA-Seq reads of each tissue, and the average mapping ratio of them.

[b] The number of protein-coding genes covered by the RNA-Seq reads of each tissue, and the total number of genes covered by at least one time.

Supplementary Table 13. Summary of Hi-C reads from the genome of *O. rehderiana*.

**Sample**	***O. rehderiana***
Raw Paired-end Reads Number	386,365,716
Clean Paired-end Reads Number	361,263,740
Clean Bases (bp)	51,506,357,437
Clean Paired-end Reads Rate	93.5%
Clean Q30 Bases Rate	92.2%

Supplementary Table 14. Mapping of Hi-C reads in the genome of *O. rehderiana*.

**Sample**	***O. rehderiana***
Clean Paired-end Reads	180,631,870
Unmapped Paired-end Reads	53,220,163
Unmapped Paired-end Reads Rate	32.3%
Paired-end Reads with Singleton	32,394,845
Paired-end Reads with Singleton Rate	17.9%
Multi Mapped Paired-end Reads	15,353,681
Multi Mapped Ratio	8.5%
Unique Mapped Paired-end Reads	74,532,069
Unique Mapped Ratio	41.3%

Supplementary Table 15. Mapping features of Hi-C reads in the genome of *O. rehderiana*.

**Sample**	***O. rehderiana***
Unique Mapped Paired-end Reads	74,532,069
Dangling End Paired-end Reads	15,870,649
Self Circle Paired-end Reads	8,737,189
Dumped Paired-end Reads	13,954,534
Valid Paired-end Reads	34,039,945
Valid Rate	45.67%

Supplementary Table 16. Summary of chromosome groups in the genome of *O. rehderiana* inferred using Hi-C data.

Chromosome groups	No. of contigs	Size of contigs (bp)
Ore1	74	65,031,946
Ore2	74	45,172,425
Ore3	64	45,030,293
Ore4	57	40,927,064
Ore5	47	39,787,669
Ore6	42	38,729,870
Ore7	55	36,919,782
Ore8	31	33,087,790
Total	444	344,686,839

Supplementary Table 17. Genome-wide distribution of different phylogenies of strictly orthologous nuclear gene groups.

Chromosomes groups	Topology I	Topology II	Topology III	Topology III / Topology II
Chr1	559	328	197	0.60
Chr2	210	133	73	0.55
Chr3	277	195	108	0.55
Chr4	280	142	113	0.80
Chr5	303	189	96	0.51
Chr6	262	164	101	0.62
Chr7	257	142	100	0.70
Chr8	266	156	118	0.76
Total	2,414	1,449	906	0.63

Supplementary references

1 Doyle, J. J. & Doyle, J. L. A rapid DNA isolation procedure for small amounts of fresh leaf tissue. *Phytochem. Bull.* **19**, 11–15 (1987).

2 Senol Cali, D., Kim, J. S., Ghose, S., Alkan, C. & Mutlu, O. Nanopore sequencing technology and tools for genome assembly: computational analysis of the current state, bottlenecks and future directions. *Brief. Bioinform.* **20**, 1542–1559 (2019).

3 Louwers, M., Splinter, E., van Driel, R., de Laat, W. & Stam, M. Studying physical chromatin interactions in plants using Chromosome Conformation Capture (3C). *Nat. Protoc.* **4**, 1216–1229 (2009).

4 Chang, S., Puryear, J. & Cairney, J. A simple and efficient method for isolating RNA from pine trees. *Plant Mol. Biol. Rep.* **11**, 113–116 (1993).

5 Bolger, A. M., Lohse, M. & Usadel, B. Trimmomatic: a flexible trimmer for Illumina sequence data. *Bioinformatics* **30**, 2114–2120 (2014).

6 Kingsford, C. A fast, lock-free approach for efficient parallel counting of occurrences of k-mers. *Bioinformatics* **27**, 764–770 (2011).

7 Walker, B. J. et al. Pilon: an integrated tool for comprehensive microbial variant detection and genome assembly improvement. *Plos One* **9**, e112963 (2014).

8 Langmead, B. & Salzberg, S. L. Fast gapped-read alignment with Bowtie 2. *Nat. Methods* **9**, 357–359 (2012).

9 Servant, N. et al. HiC-Pro: an optimized and flexible pipeline for Hi-C data processing. *Genome Biol.* **16**, 259 (2015).

10 Burton, J. N. et al. Chromosome-scale scaffolding of *de novo* genome assemblies based on chromatin interactions. *Nat. Biotechnol.* **31**, 1119–1125 (2013).

11 Chen, N. Using RepeatMasker to identify repetitive elements in genomic sequences. *Curr. Protoc. Bioinf.* **5**, 4.10.1–4.10.14 (2004).

12 Bao, W., Kojima, K. K. & Kohany, O. Repbase update, a database of repetitive elements in eukaryotic genomes. *Mobile DNA* **6**, 11 (2015).

13 Grabherr, M. G. et al. Full-length transcriptome assembly from RNA-Seq data without a reference genome. *Nat. Biotechnol.* **29**, 644–652 (2011).

14 Haas, B. J. et al. Improving the *Arabidopsis* genome annotation using maximal transcript alignment assemblies. *Nucleic Acids Res.* **31**, 5654–5666 (2003).

15 Stanke, M. et al. AUGUSTUS: *ab initio* prediction of alternative transcripts. *Nucleic Acids Res.* **34**, W435–W439 (2006).

16 Kim, D., Langmead, B. & Salzberg, S. L. HISAT: a fast spliced aligner with low memory requirements. *Nat. Methods* **12**, 357–360 (2015).

17 Arabidopsis Genome Initiative. Analysis of the genome sequence of the flowering plant *Arabidopsis thaliana*. *Nature* **408**, 796–815 (2000).

18 Salojärvi, J. et al. Genome sequencing and population genomic analyses provide insights into the adaptive landscape of silver birch. *Nat. Genet.* **49**, 904–912 (2017).

19 Yang, Y. Z. et al. Genomic effects of population collapse in a critically endangered ironwood tree *Ostrya rehderiana*. *Nat. Commun.* **9**, 5449 (2018).

20 Yang, X. Y. et al. A chromosome-level reference genome of the hornbeam, *Carpinus fangiana*. *Sci. Data* **7**, 24 (2020).

21 Camacho, C. et al. BLAST+: architecture and applications. *BMC Bioinf.* **10**, 421 (2009).

22 Birney, E., Clamp, M. & Durbin, R. GeneWise and Genomewise. *Genome Res.* **14**, 988–995 (2004).

23 Haas, B. J. et al. Automated eukaryotic gene structure annotation using EVidenceModeler and the Program to Assemble Spliced Alignments. *Genome Biol.* **9**, R7 (2008).

24 Bairoch, A. & Apweiler, R. The SWISS-PROT protein sequence database and its supplement TrEMBL in 2000. *Nucleic Acids Res.* **28**, 45–48 (2000).

25 Marchler-Bauer, A. et al. CDD: a Conserved Domain Database for the functional annotation of proteins. *Nucleic Acids Res.* **39**, D225–D229 (2011).

26 Hunter, S. et al. InterPro: the integrative protein signature database. *Nucleic Acids Res.* **37**, D211–D215 (2009).

27 Ashburner, M. et al. Gene Ontology: tool for the unification of biology. *Nat. Genet.* **25**, 25–29 (2000).

28 Conesa, A. & Götz, S. Blast2GO: a comprehensive suite for functional analysis in plant genomics. *Int. J. Plant Genomics* **2008**, 619832 (2008).

29 Moriya, Y., Itoh, M., Okuda, S., Yoshizawa, A. C. & Kanehisa, M. KAAS: an automatic genome annotation and pathway reconstruction server. *Nucleic Acids Res.* **35**, W182–W185 (2007).

30 Simão, F. A., Waterhouse, R. M., Ioannidis, P., Kriventseva, E. V. & Zdobnov, E. M. BUSCO: assessing genome assembly and annotation completeness with single-copy orthologs. *Bioinformatics* **31**, 3210–3212 (2015).

31 Kriventseva, E. V. et al. OrthoDB v10: sampling the diversity of animal, plant, fungal, protist, bacterial and viral genomes for evolutionary and functional annotations of orthologs. *Nucleic Acids Res.* **47**, D807–D811 (2019).

32 Wang, Y. P. et al. MCScanX: a toolkit for detection and evolutionary analysis of gene synteny and collinearity. *Nucleic Acids Res.* **40**, e49 (2012).

33 Krzywinski, M. et al. Circos: an information aesthetic for comparative genomics. *Genome Res.* **19**, 1639–1645 (2009).

34 Wang, Z. F. et al. Hybrid speciation via inheritance of alternate alleles of parental isolating genes. *Mol. Plant* **14**, 208–222 (2021).

35 Li, Y. et al. The *Corylus mandshurica* genome provides insights into the evolution of Betulaceae genomes and hazelnut breeding. *Hortic. Res.* **8**, 54 (2021).

36 Ye, G. et al. *De novo* genome assembly of the stress tolerant forest species *Casuarina equisetifolia* provides insight into secondary growth. *Plant J.* **97**, 779–794 (2019).

37 Marrano, A. et al. High-quality chromosome-scale assembly of the walnut (*Juglans regia* L.) reference genome. *GigaScience* **9**, giaa050 (2020).

38 Emms, D. M. & Kelly, S. OrthoFinder: phylogenetic orthology inference for comparative genomics. *Genome Biol.* **20**, 238 (2019).

39 Löytynoja, A. *Phylogeny-aware alignment with PRANK*. In: *Multiple Sequence Alignment Methods, Methods in Molecular Biology* (Humana Press, 2014).

40 Stamatakis, A. RAxML version 8: a tool for phylogenetic analysis and post-analysis of large phylogenies. *Bioinformatics* **30**, 1312–1313 (2014).

41 Kielbasa, S. M., Wan, R., Sato, K., Horton, P. & Frith, M. C. Adaptive seeds tame genomic sequence comparison. *Genome Res.* **21**, 487–493 (2011).

42 Sayyari, E. & Mirarab, S. Fast coalescent-based computation of local branch support from quartet frequencies. *Mol. Biol. Evol.* **33**, 1654–1668 (2016).

43 Sukumaran, J. & Holder, M. T. DendroPy: a Python library for phylogenetic computing. *Bioinformatics* **26**, 1569–1571 (2010).

44 Friis, E. M., Pedersen, K. R. & Schonenberger, J. *Endressianthus*, a new normapolles-producing plant genus of fagalean affinity from the Late Cretaceous of Portugal. *Int. J. Plant Sci.* **164**, S201–S223 (2003).

45 Yang, X. Y. et al. Plastomes of Betulaceae and phylogenetic implications. *J. Syst. Evol.* **57**, 508–518 (2019).

46 Li, H. & Durbin, R. Fast and accurate short read alignment with Burrows-Wheeler transform. *Bioinformatics* **25**, 1754–1760 (2009).

47 McKenna, A. et al. The Genome Analysis Toolkit: a MapReduce framework for analyzing next-generation DNA sequencing data. *Genome Res.* **20**, 1297–1303 (2010).

48 Rokas, A. & Holland, P. Rare genomic changes as a tool for phylogenetics. *Trends Ecol. Evol.* **15**, 454–459 (2000).

49 Bapteste, E. & Philippe, H. The potential value of indels as phylogenetic markers: position of trichomonads as a case study. *Mol. Biol. Evol.* **19**, 972–977 (2002).

50 Jiang, Y. F. et al. Differentiating homoploid hybridization from ancestral subdivision in evaluating the origin of the D lineage in wheat. *New Phytol.* **228**, 409–414 (2020).

51 Blischak, P. D., Chifman, J., Wolfe, A. D. & Kubatko, L. S. HyDe: a Python package for genome-scale

hybridization detection. *Syst. Biol.* **67**, 821–829 (2018).

52 Dasmahapatra, K. K. et al. Butterfly genome reveals promiscuous exchange of mimicry adaptations among species. *Nature* **487**, 94–98 (2012).

53 Green, R. E. et al. A draft sequence of the Neandertal genome. *Science* **328**, 710–722 (2010).

54 Durand, E. Y., Patterson, N., Reich, D. & Slatkin, M. Testing for ancient admixture between closely related populations. *Mol. Biol. Evol.* **28**, 2239–2252 (2011).

55 Patterson, N. et al. Ancient admixture in human history. *Genetics* **192**, 1065–1093 (2012).

56 Malinsky, M., Matschiner, M. & Svardal, H. Dsuite - Fast *D*-statistics and related admixture evidence from VCF files. *Mol. Ecol. Resour.* **21**, 584–595 (2021).

57 Hudson, R. R., Kreitman, M. & Aguadé, M. A test of neutral molecular evolution based on nucleotide data. *Genetics* **116**, 153–159 (1987).
