# Supplementary material for: Genomic evidence for homoploid hybrid speciation between ancestors of two different genera
Source: Nat Commun. 2022 Apr 13;13:1987. doi: 10.1038/s41467-022-29643-4 (PMC9008057; doi:10.1038/s41467-022-29643-4)
Supplement: Supplementary file 3 — Description of Additional Supplementary Files [file 41467_2022_29643_MOESM3_ESM.pdf]

Description of Additional Supplementary Files

File Name: Supplementary Data 1
Description: Summary of studies on HHS and introgression (from 2012 to 2021) based on population genomic data.

File Name: Supplementary Data 2
Description: . Individuals sampled in this study and their locations.

File Name: Supplementary Data 3
Description: Assessment of different reference genomes using randomly selected samples.

File Name: Supplementary Data 4
Description: Genome-resequencing statistics for the individuals sampled in this study.

File Name: Supplementary Data 5
Description: PSGs identified in sect. Distegocarpus derived from sect. Carpinus.

File Name: Supplementary Data 6
Description: PSGs identified in sect. Distegocarpus derived from Ostrya.

File Name: Supplementary Data 7
Description: Genes identified as harboring hybrid signals.
