# Supplementary material for: Genomic evidence for homoploid hybrid speciation between ancestors of two different genera
Source: Nat Commun. 2022 Apr 13;13:1987. doi: 10.1038/s41467-022-29643-4 (PMC9008057; doi:10.1038/s41467-022-29643-4)
Supplement: Supplementary file 11 — Reporting Summary [file 41467_2022_29643_MOESM11_ESM.pdf]

nature portfolio

Corresponding author(s): Jianquan Liu

Last updated by author(s): Mar 19, 2022

Reporting Summary

Nature Portfolio wishes to improve the reproducibility of the work that we publish. This form provides structure for consistency and transparency in reporting. For further information on Nature Portfolio policies, see our Editorial Policies and the Editorial Policy Checklist.

Statistics

For all statistical analyses, confirm that the following items are present in the figure legend, table legend, main text, or Methods section.

n/a	Confirmed	
☐	☒	The exact sample size (*n*) for each experimental group/condition, given as a discrete number and unit of measurement
☐	☒	A statement on whether measurements were taken from distinct samples or whether the same sample was measured repeatedly
☐	☒	The statistical test(s) used AND whether they are one- or two-sided<br>*Only common tests should be described solely by name; describe more complex techniques in the Methods section.*
☒	☐	A description of all covariates tested
☐	☒	A description of any assumptions or corrections, such as tests of normality and adjustment for multiple comparisons
☐	☒	A full description of the statistical parameters including central tendency (e.g. means) or other basic estimates (e.g. regression coefficient) AND variation (e.g. standard deviation) or associated estimates of uncertainty (e.g. confidence intervals)
☐	☒	For null hypothesis testing, the test statistic (e.g. *F*, *t*, *r*) with confidence intervals, effect sizes, degrees of freedom and *P* value noted<br>*Give P values as exact values whenever suitable.*
☒	☐	For Bayesian analysis, information on the choice of priors and Markov chain Monte Carlo settings
☒	☐	For hierarchical and complex designs, identification of the appropriate level for tests and full reporting of outcomes
☒	☐	Estimates of effect sizes (e.g. Cohen's *d*, Pearson's *r*), indicating how they were calculated

Our web collection on statistics for biologists contains articles on many of the points above.

Software and code

Policy information about availability of computer code

Data collection	Data collection has been described in the Methods and Supplementary Information. No software has been used for data collection
Data analysis	Software used are listed as follows: Guppy (v3.2.2), Trimmomatic (v0.36), Jellyfish (v2.2.9), NextDenovo (v2.0), SmartDenovo (v1.0.0), Pilon (v1.22), bowtie2 (v2.3.2), HiC-Pro (v3.1.0), LACHESIS (v1), RepeatMasker (v4.0.7), RepeatModeler (v1.0.10), PASA pipeline (v2.1.0), Augustus (v3.2.3), HISAT2 (v2.1.0), TBLASTN (v2.2.31), GeneWise (v2.4.1), EVidenceModeler (v1.1.1), BLAST+ (v2.2.31), InterProScan (v5.25-64.0), Blast2GO (v4.1), BUSCO (v3), MCScanX (v1), Circos (v0.69), OrthoFinder (v2.3.8), PRANK (v170427), RAxML (v8.2.12), LAST (v963), R (v4.1.2), ASTRAL (v5.6.3), DendroPy (v4.2.0), BWA (v0.7.17), SAMtools (v1.5), GATK (v3.8), HyDe (v0.4.3), and Dsuit (v0.4).

For manuscripts utilizing custom algorithms or software that are central to the research but not yet described in published literature, software must be made available to editors and reviewers. We strongly encourage code deposition in a community repository (e.g. GitHub). See the Nature Portfolio guidelines for submitting code & software for further information.

Data

Policy information about availability of data

All manuscripts must include a data availability statement. This statement should provide the following information, where applicable:

- Accession codes, unique identifiers, or web links for publicly available datasets
- A description of any restrictions on data availability
- For clinical datasets or third party data, please ensure that the statement adheres to our policy

All the sequencing data have been deposited in the National Genomics Data Center (https://ngdc.cncb.ac.cn). The sequencing data for the de novo genome of Carpinus viminea (including the ONT long reads, Illumina reads of WGS, Hi-C reads, and transcriptomes) have been deposited under the accession number PRJCA005724. The Hi-C sequencing data for Ostrya rehderiana have been deposited under the accession number PRJCA005717. The resequencing data for all

individuals have been deposited under the accession numbers PRJCA003130 and PRJCA005842. The genome assemblies and annotations of C. viminea and O. rehderiana have been uploaded to figshare (https://doi.org/10.6084/m9.figshare.14988777). The SwissProt and TrEMBL databases used in this study are available from https://www.uniprot.org. KEGG PATHWAY database can be available from https://www.kegg.jp. There is no restriction on data availability. Source data are provided with this paper.

Field-specific reporting

Please select the one below that is the best fit for your research. If you are not sure, read the appropriate sections before making your selection.

☐ Life sciences ☐ Behavioural & social sciences ☒ Ecological, evolutionary & environmental sciences

For a reference copy of the document with all sections, see nature.com/documents/nr-reporting-summary-flat.pdf

Ecological, evolutionary & environmental sciences study design

All studies must disclose on these points even when the disclosure is negative.

Study description	We generated a chromosome-level reference genome of Carpinus viminea of sect. Carpinus and re-sequenced genomes of 47 individuals from the genera Carpinus, Ostrya, and Ostryopsis (as outgroup) in Betulaceae. Our integrated analyses together suggested that sect. Distegocarpus with three species originated through HHS during the early divergence between Carpinus and Ostrya.
Research sample	Species names, population codes, sample locations, and sample sizes for whole genome resequencing of individuals are described in Supplementary Data 2. All were adult individuals collected in the wild. Within each population, sampling was performed randomly to ensure representative samples.
Sampling strategy	We collected a wild plant of Carpinus viminea for de novo genome sequencing and a wild plant of Ostrya rehderiana for Hi-C sequencing. We collected 47 individuals (including a total of 21 species) from three different lineages (and outgroup) for population genomic re-sequencing: 10 individuals (from 10 species) of sect. Carpinus, 27 individuals (from 3 species) of sect. Distegocarpus, 7 individuals (from 7 species) of Ostrya, and 3 individuals (from 1 species) of Ostryopsis as outgroup. Except for the outgroup, all the sampled individuals were selected from different populations (one individual per population). The samples of sect. Carpinus and Ostrya covered all the major species of these two lineages. The samples of sect. Distegocarpus covered all 3 species in this lineage and almost the whole of their distributions in the wild.
Data collection	Samples were collected in the wild by C.C. Sampling sites were positioned by GPS and recorded in a laptop. DNA was prepared by Z.W. Genome sequencing data were generated by different types of sequencers.
Timing and spatial scale	Spatial scale of sampling for the study is described in Supplementary Data 2 (spanning USA, Slovenia, Russia, and China). All samples were collected between 2016-2020.
Data exclusions	No data were excluded.
Reproducibility	All sequencing for genome assembly were more than 100 fold coverage. For each of the 47 re-sequenced samples, an average of more than 25 fold coverage clean bases were sequenced. All phylogenetic analyses were performed with 100 bootstraps.
Randomization	Samples were randomized for collection.
Blinding	Blinding is not applicable in our study because it does not involve subjects which receive different treatments.

Did the study involve field work? ☐ Yes ☒ No

Reporting for specific materials, systems and methods

We require information from authors about some types of materials, experimental systems and methods used in many studies. Here, indicate whether each material, system or method listed is relevant to your study. If you are not sure if a list item applies to your research, read the appropriate section before selecting a response.

Materials & experimental systems

n/a	Involved in the study
☒	☐ Antibodies
☒	☐ Eukaryotic cell lines
☒	☐ Palaeontology and archaeology
☒	☐ Animals and other organisms
☒	☐ Human research participants
☒	☐ Clinical data
☒	☐ Dual use research of concern

Methods

n/a	Involved in the study
☒	☐ ChIP-seq
☒	☐ Flow cytometry
☒	☐ MRI-based neuroimaging
